# Inhalable Protein Powder Prepared by Spray-Freeze-Drying Using Hydroxypropyl-β-Cyclodextrin as Excipient

**DOI:** 10.3390/pharmaceutics13050615

**Published:** 2021-04-24

**Authors:** Jason C. K. Lo, Harry W. Pan, Jenny K. W. Lam

**Affiliations:** 1 Department of Pharmacology and Pharmacy, Li Ka Shing Faculty of Medicine, The University of Hong Kong, 21 Sassoon Road, Pokfulam, Hong Kong, China; lckjason@connect.hku.hk (J.C.K.L.); hwpan@connect.hku.hk (H.W.P.); 2 Advanced Biomedical Instrumentation Centre, Hong Kong Science Park, Shatin, New Territories, Hong Kong, China

**Keywords:** aerosolization, cyclodextrin, factorial design, inhalation, protein delivery, pulmonary delivery, spray-freeze-drying

## Abstract

The prospect of inhaled biologics has garnered particular interest given the benefits of the pulmonary route of administration. Pertinent considerations in producing inhalable dry powders containing biological medicines relate to aerosol performance and protein stability. Spray-freeze-drying (SFD) has emerged as an established method to generate microparticles that can potentially be deposited in the lungs. Here, the SFD conditions and formulation composition were evaluated using bovine serum albumin (BSA) as a model protein and 2-hydroxypropyl-beta-cyclodextrin (HPβCD) as the protein stabilizer. A factorial design analysis was performed to investigate the effects of BSA content, solute concentration of feed solution, and atomization gas flow rate on dispersibility (as an emitted fraction), respirability (as fine particle fraction), particle size, and level of protein aggregation. The atomization gas flow rate was identified as a significant factor in influencing the aerosol performance of the powder formulations and protein aggregation. Nonetheless, high atomization gas flow rate induced aggregation, highlighting the need to further optimize the formulation. Of note, all the formulations exhibited excellent dispersibility, while no fragmentation of BSA occurred, indicating the feasibility of SFD and the promise of HPβCD as an excipient.

## 1. Introduction

Inhaled protein therapy has attracted great attention following the rapidly expanding biological candidates to treat a range of respiratory diseases [1,2,3]. Pulmonary delivery offers a non-invasive way to deliver drugs directly to the lung of patients with a lower required dose compared to the systemic route. The dry powder has been in the spotlight for inhaled protein formulations thanks to better stability and longer shelf life.

The formulation of inhalable protein powders poses significant challenges. Spray-drying (SD) is a common method to prepare dry powders as the process is highly controllable. However, it also suffers from disadvantages, such as high-temperature exposure with relatively low production yield [4]. Spray-freeze-drying (SFD) is another particle engineering technique that has become increasingly popular in producing dry powders for pulmonary delivery of a wide range of therapeutic molecules, including biologics and macromolecules such as proteins, bacteriophages, and nucleic acids [5,6,7,8,9]. Unlike SD, no heating is required for the dehydration process in SFD. Typically, a liquid formulation is fed into a nozzle, and the atomized droplets are instantaneously frozen in a cold atmosphere or cryogenic liquid, such as liquid nitrogen. Finally, the frozen solvent is sublimed and removed under a near-vacuum [10]. The low temperature throughout the production process may be favorable to protein therapeutics, which are usually heat-sensitive, and the production yield is generally better than SD. More importantly, the highly porous and spherical spray-freeze-dried particles that are low in density usually exhibit good aerosol behavior [9]. However, inevitably, proteins are still exposed to shear stress during the atomization. The instant freezing and subsequent drying of a protein may also contribute to thermodynamic instability and induce protein aggregation or degradation. Hence, stabilizing excipients must be added to the formulation. Because of the multiple stresses encountered, a combination of excipients is usually applied to the protein formulation in SFD. Polyols (such as mannitol), sugars (such as lactose and trehalose) and surfactants (such as polysorbates 20 and 80) are commonly used in dry powder formulations [11,12,13], and their uses are mainly investigated in SD (where shear and thermal stresses dominate) and freeze-drying (stresses from lyophilization).

Cyclodextrin is an oligosaccharide that is known for its ability to enhance the solubilization of poorly water-soluble drugs and to protect macromolecules in liquid and solid states [14]. It protects proteins in the dried form by various mechanisms, including water replacement, vitrification, amino acid complexation, and surfactant-like effect [15,16,17,18]. 2-hydroxypropyl-β-cyclodextrin (HPβCD) is a hydroxyalkyl derivative of cyclodextrin. It is an ideal candidate for its stabilization effect due to the considerable availability of hydrogen bonds that consolidates its role in water replacement. Its non-hygroscopic nature confers an extra advantage over other sugars like trehalose and lactose in preventing moisture absorption and, therefore, maintaining powder dispersibility [19]. With its good safety profile, it has great potential to be used as an excipient in protein dry powder formulations as both a stabilizing and a bulking agent.

Excellent aerosol performance and protein stability are crucial in successfully developing inhaled dry powders of protein therapeutics. Formulations and production methods must be controlled and optimized to maintain an adequate balance between the two criteria. In this study, bovine serum albumin (BSA) was employed as a model protein for dry powder preparation by SFD. The relatively low molecular weight of BSA may provide some insights into the formulation of biologics of similar molecular weights, such as antigen-binding fragments (Fab), notwithstanding conditions that need to be optimized for each biological entity in product development. Three factors were selected for optimization: (i) protein content; (ii) solute concentration of the feed solution; and (iii) atomization gas flow rate. Through using a factorial design approach, this study aimed to examine the effects of these three factors systematically on the aerosol property and protein aggregation in spray-freeze-dried powders and to fill the unknown gap in the potential of HPβCD as an excipient and the major constituent of a protein formulation for use in dry powder inhalers.

## 2. Materials and Methods

### 2.1. Materials

BSA (M_w_ 66 kg/mol), HPβCD (average M_w_ ~ 1540 g/mol), sodium phosphate, and Coomassie brilliant blue R were purchased from Sigma-Aldrich (St. Louis, MO, USA). Bradford protein assay dye reagent and acrylamide were acquired from Bio-Rad Laboratories (Hercules, CA, USA). Glacial acetic acid and ortho-phosphoric acid were obtained from Merck KGaA (Darmstadt, Germany). PageRuler™ prestained protein ladder (10 to 180 kDa) was purchased from Thermo Scientific (Waltham, MA, USA). Methanol was obtained from Anaqua Global International (Cleveland, OH, USA). Ultrapure water used was purified by Barnstead NANOpure Diamond™ water system with a 0.2 µm filter (APS Water Services, Van Nuys, CA, USA). All solvents and reagents were of analytical grade or better unless otherwise specified.

### 2.2. Design of Experiment by Factorial Design

A three-factor two-level (2^3^) full factorial design was employed to design the spray-freeze-dried powder formulations (Table 1). The investigated factors were: A—BSA content (the percentage of BSA in the solute; % *w*/*w*); B—solute concentration (concentration of total solute, BSA plus HPβCD; % *w*/*v*); and C—atomization gas flow rate (L/h). The levels of each variable were designated as −1, 0 and +1. The levels of center point were set midway between the high and low levels. The center point formulation was prepared in triplicate to evaluate the variability of the formulation model. After the optimal condition was identified, five extended formulations were prepared to further investigate the effects of BSA content in the formulation.

### 2.3. Dry Powder Preparation by Spray-Freeze-Drying (SFD)

A total of 16 formulations were prepared, 11 according to the factorial design plus five extended formulations (Table 2). The feed solutions for SFD were prepared by mixing appropriate volumes of BSA and HPβCD stock solutions with ultrapure water to achieve a total solute mass of 120 mg. Stock solutions of BSA and HPβCD for the factorial formulations were prepared at 15 mg/mL and 150 mg/mL, respectively, while stock solutions of BSA and HPβCD for the extended formulations were prepared at 100 mg/mL. For the SFD step, the feed solution was first drawn into a 10 mL syringe (Terumo Corporation, Tokyo, Japan), which was connected via a tube to a two-fluid nozzle (Büchi Labortechnik AG, Flawil, Switzerland) of 0.7 mm internal diameter for atomization. The nozzle was positioned above a stainless-steel tank containing liquid nitrogen to facilitate instantaneous freezing [7]. The nitrogen gas flow rate for atomization was set according to the factorial design. The feed solution was then fed into the nozzle at a controlled feed rate of 2 mL/min using a syringe pump (Legato^™^ 210, KD Scientific, Holliston, MA, USA). The atomized liquid droplets were immediately frozen as they traveled towards the liquid nitrogen. Primary drying was conducted in a freeze-dryer (FreeZone^®^ 6 Liter benchtop freeze-dry system with stoppering tray dryer, Labconco Corporation, Missouri, MO, USA) at a chamber pressure below 0.14 mbar at −25 °C for 20 h. Following this, the temperature was gradually increased to 20 °C over 4 h and thereafter kept constant for at least another 40 h to allow secondary drying. The dried powders were collected and stored in a desiccator with silica gel (10% humidity as monitored) at ambient temperature until further analysis. The production yield was calculated as the percentage of the mass of powder collected to the initial solute mass input, assuming negligible moisture content in the collected powder.

### 2.4. Quantification of HPβCD and BSA in Spray-Freeze-Dried Powder

The proportion of HPβCD and/or BSA in the spray-freeze-dried powder of each sample was measured. For each formulation, 4 mg of powder was weighed and dissolved in ultrapure water to a final volume of 5 mL. The samples were filtered through a 0.45 µm nylon syringe filter before quantifying the BSA and/or HPβCD concentration by HPLC, as described below. The experiment was performed in triplicate.

### 2.5. High-Performance Liquid Chromatography (HPLC) and Size Exclusion Chromatography (SEC)

HPβCD was detected by HPLC (Agilent Technologies 1260 Infinity Series, Santa Clara, CA, USA) with a refractive index detector. Two Agilent Hi-Plex H guard columns (50 × 7.7 mm, 8 µm) were connected following an Agilent Hi-Plex H guard cartridge (5 × 3 mm, 8 µm). Ultrapure water was used as the mobile phase running at an isocratic flow rate of 0.6 mL/min. The column temperature was controlled at 65 °C. A volume of 50 µL was injected, followed by a running time of 8.5 min per sample. HPβCD was quantified as the area under the curve of the refractive index signal and quantified against a standard curve ranging from 7.8125 to 1000 µg/mL with a retention time of the peak at around 2.9 min. BSA was detected by SEC-HPLC using a diode array detector with detection at 214 nm. A 300 × 7.8 mm LC column (Yarra™ 3 µm SEC-3000, Phenomenex^®^, Torrance, CA, USA) was used. The mobile phase consisted of 0.15 M sodium phosphate (pH 6.8) and was run at an isocratic flow rate of 0.8 mL/min. The column was maintained at a controlled temperature of 25 °C. A volume of 100 µL was injected, followed by a running time of 18 min per sample. BSA was quantified as the area under the curve of the chromatogram and quantified against a standard curve ranging from 40 to 800 µg/mL with the retention time of the peak for BSA monomer at around 11.1 min. The peak retention time was similar for the unsprayed BSA and the reconstituted spray-freeze-dried BSA. The level of protein aggregation was calculated by dividing the area of the monomer peak by the total area integrated.

### 2.6. Particle Morphology by Scanning Electron Microscopy (SEM)

The morphology of the spray-freeze-dried particles was analyzed by field emission scanning electron microscope (Hitachi S-4800, Tokyo, Japan) at 5.0 kV. The powder was affixed on a double-sided carbon tape to an SEM aluminum stub. The powder samples were sputter-coated with approximately 4 nm iridium in two 60 s cycles by a sputter-coater (SCD 005, BAL-TEC GmbH, Schalksmühle, Germany) to avoid charging during imaging.

### 2.7. Particle Size Distribution Measurement by Laser Diffractometry

The volumetric size distribution of the spray-freeze-dried powders was evaluated by laser diffraction. A laser diffractometer configured with a dosing unit for inhalers and nebulizers (HELOS/KR+INHALER, Sympatec GmbH, Clausthal-Zellerfeld, Germany) was used to determine the size distribution of the particles dispersed from a Breezhaler^®^ (Novartis AG, Basel, Switzerland) as previously described [20]. In brief, the Breezhaler^®^ was mounted onto the central unit of the INHALER module by inserting the mouthpiece into the adaptor horizontally. A suction airflow was provided by a vacuum pump at 60 L/min. Prior to measurement, a size 3 gelatin capsule (Capsugel^®^, Morristown, NJ, USA) was loaded with 1.0 ± 0.1 mg powder and placed in a Breezhaler^®^. The particle size distribution was calculated by the WINDOX 5 software (version 5.8.0.0, Sympatec GmbH, Clausthal-Zellerfeld, Germany) based on the enhanced Fraunhofer theory. Particle size data were expressed as D_10_, D_50_, and D_90_, which represent the equivalent spherical volume diameters at 10%, 50%, and 90% cumulative volumes, respectively. Span was calculated as (D_90_–D_10_)/D_50_. The experiment was performed in triplicate.

### 2.8. Aerosol Performance by Next-Generation Impactor (NGI)

The aerosol performance of the spray-freeze-dried powders was evaluated with an NGI (Copley Scientific, Nottingham, UK) as previously described [20]. For each dispersion of the factorial design formulations, 3.5 ± 0.1 mg of powder was encapsulated in a size 3 gelatin capsule. For each dispersion of the extended formulations, a total of 16.8 ± 0.1 mg powder was encapsulated into three sizes 3 gelatin capsules so that the amount of protein was sufficient to be quantified by SEC-HPLC. The capsule was pierced in a Breezhaler^®^. Prior to each dispersion, a thin layer of silicone grease (LPS^®^ Dry Film Silicone Lubricant, Tucker, GA, USA) was coated onto the impactor collection cups to reduce particle bounce. Powder dispersion into the NGI was carried out with 4 L of air drawn into the NGI. All formulations were dispersed at 60 L/min using a Breezhaler^®^ for 4.0 s. After dispersion, ultrapure water was used to dissolve and rinse the samples from all stages—3.5 mL for the capsule, inhaler, adaptor, NGI stages 2 to 7, and micro-orifice collector (MOC); and 5 mL for the induction port (throat) and stage 1. Powder dispersions were performed in triplicate for each formulation. The mass of HPβCD deposited in each stage was quantified by HPLC with refractive index detection as described. The mass of BSA (for extended formulations, except EXT-0) was quantified by SEC-HPLC as mentioned above. The emitted fraction (EF) is defined as the fraction of powder that exited the inhaler concerning the recovered dose. Fine particle dose (FPD) is defined as the mass of particles with an aerodynamic diameter of less than 5.0 µm. Fine particle fraction (FPF) is defined as the percentage fraction of FPD concerning the recovered dose. The mass median aerodynamic diameter (MMAD) is defined as the aerodynamic diameter at which half of the particles by mass are larger and the other half smaller. The MMAD and the geometric standard deviation (GSD) were calculated based on the NGI results with reference to the United States Pharmacopoeia (USP) <601>.

### 2.9. Protein Integrity by Sodium Dodecyl Sulfate-Polyacrylamide Gel Electrophoresis (SDS-PAGE)

Protein integrity was evaluated using nonreducing SDS-PAGE. The electrophoresis was prepared using a MiniPROTEAN^®^ tetra hand-cast system (Bio-Rad Laboratories, Hercules, CA, USA). The spray-freeze-dried powders were dissolved in water. A control showing BSA degradation was prepared by heating 1 mg/mL BSA in pH 2.0 phosphate-buffered saline (PBS) at 60 °C for 4 h. A control showing BSA aggregation was prepared by heating 1 mg/mL BSA in pH 7.4 PBS at 60 °C for 4 h. A sample containing 5 µg BSA was loaded into each well and electrophoresed in a 10% polyacrylamide gel. The gel was then stained with Coomassie brilliant blue R solution for 1.5 h and subsequently destained with methanol-glacial acetic acid-distilled water (5:1:4, *v*/*v*) solution, both at ambient temperature on an orbital shaker. The next day, the gel was rinsed with distilled water for 2 h on the orbital shaker for rehydration and photographed using a G:BOX Chemi XR5 imaging system (Syngene, Cambridge, UK).

### 2.10. Statistical Analysis

Minitab^®^ 18.1 statistical package software (Minitab LLC, State College, PA, USA) was used for the design of experiment and factorial analysis in the form of analysis of variance (ANOVA). The analyzed response variables included EF, FPF, median volumetric particle size and protein aggregation. Any term that crosses the reference line in the factorial analysis was considered statistically significant. Unless stated otherwise, all other experimental results were analyzed by either one-way ANOVA followed by Tukey’s post hoc test or Student’s *t*-test, whichever appropriate, using Prism 7 (version 7.02, GraphPad software, San Diego, CA, USA). A significance level of α = 0.05 was selected throughout this study.

## 3. Results

### 3.1. Production Yield and Composition

The extended formulations had slightly higher production yields compared with the factorial design formulations (Table 3). This could be attributed to the higher solute mass in the feed solution (200 mg vs. 120 mg) and higher concentration of the BSA stock solution that was used to prepare the feed solution (100 mg/mL vs. 15 mg/mL) than those of the factorial design formulations. The composition of the spray-freeze-dried formulations was analyzed by measuring the BSA and/or HPβCD content in the formulations. Only HPβCD was measured in the factorial design formulations due to the relatively low content of protein (below 10%). Both BSA and HPβCD were measured in the extended formulations, except EXT-0 (which did not contain any BSA) and EXT-100 (which did not contain any HPβCD). Across all the formulations, the theoretical and measured contents of BSA and HPβCD were within 5% deviation, suggesting that the composition of the spray-freeze-dried powders was similar to the mass of input.

### 3.2. Particle Morphology and Size Distribution

**Figure 1:**
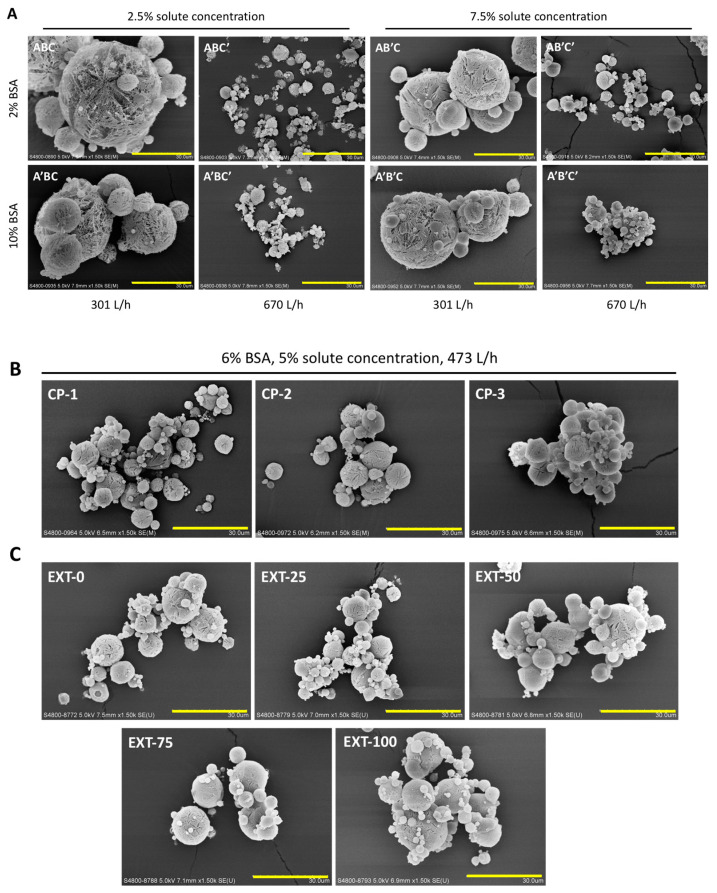
Scanning electron microscope (SEM) images of spray-freeze-dried powders. (**A**) The eight formulations prepared according to the factorial design; (**B**) the three center point formulations; (**C**) the five extended formulations. Scale bar: 30 µm.

The morphology of the spray-freeze-dried powders was visualized by SEM, and all powders presented spherical, porous structures (Figure 1). In general, the particle size decreased as the atomization flow rate increased. It was observed that the particles became more porous when the solute concentration decreased. There was no noticeable difference in morphology between particles prepared under the same operating conditions (i.e., same atomization flow rate and solute concentration), but different concentrations of BSA, although the particles containing higher BSA content tend to be more aggregative, as demonstrated in A’BC’ and A’B’C’ (compared to ABC’ and AB’C’, respectively). The volumetric size distribution of the airflow-dispersed spray-freeze-dried powders was measured by laser diffraction (Table 4). Consistent with the trend shown in the SEM images, particles prepared at a higher atomization flow rate exhibited a smaller particle size.

### 3.3. Aerosol Performance

The aerosol performance of the spray-freeze-dried powders was evaluated with an NGI. The data were presented as EF, FPF and MMAD (Table 4). All the EFs were over 90%, and the differences between the formulations were small. In the factorial design formulations, ABC’ and A’BC’ had the highest FPF of 78.4% and 79.4%, respectively, with MMAD below 1 µm. Both powders were produced at a low solute concentration (2.5% *w*/*v*) and high atomization gas flow rate (670 L/h). Five formulations (AB’C’, A’B’C’, CP1—3) displayed MMAD between 1 and 5 µm, and their FPFs were around 60%, with no significant differences between them.

### 3.4. Protein Integrity and Aggregation

**Figure 2:**
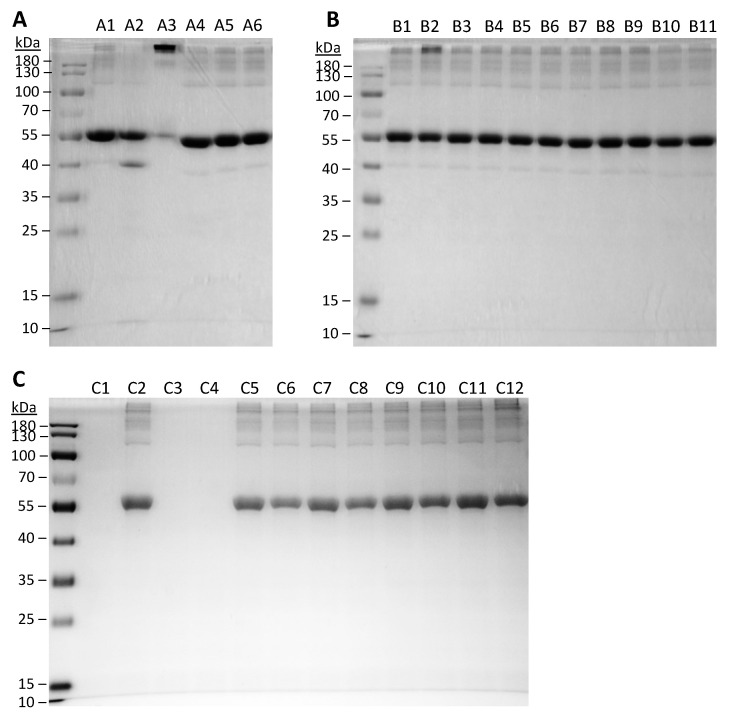
Integrity of BSA protein as examined by gel electrophoresis (SDS-PAGE). (**A**) Samples of controls without undergoing spray-freeze-drying (SFD). A1, unprocessed BSA; A2, BSA treated in pH 2 at 60 °C (degradation control); A3, BSA treated in pH 7 at 60 °C (aggregation control); physical mixtures of BSA-HPβCD with 2% BSA (A4), 6% BSA (A5), and 10% BSA (A6). (**B**) Factorial design formulations. B1–8, in the same running order as in Table 3 and Table 4; B9–11, CP-1–3, respectively. (**C**) Extended formulations and control samples. C1, unprocessed HPβCD; C2, unprocessed BSA; EXT-0 before (C3) and after (C4) SFD; EXT-25 before (C5) and after (C6) SFD; EXT-50 before (C7) and after (C8) SFD; EXT-75 before (C9) and after (C10) SFD; EXT-100 before (C11) SFD and after (C12) SFD.

**Figure 3:**
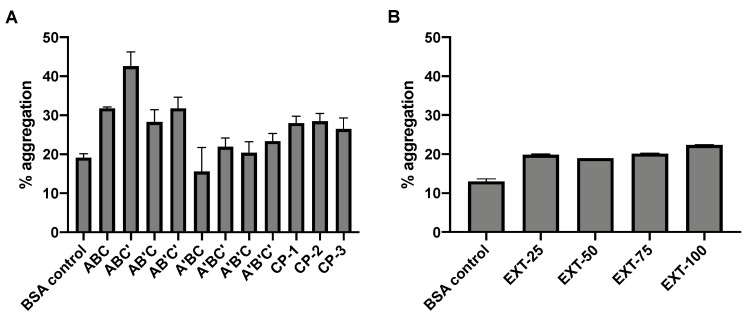
Protein aggregation of (**A**) factorial formulations and (**B**) extended formulations as determined by size exclusion chromatography (SEC). BSA control (unprocessed protein) was included as control. Data are presented as mean ± standard deviation (*n* = 3).

The molecular weight of BSA following SFD was examined by SDS-PAGE, which provides information about the stability of the protein in terms of aggregation and degradation (Figure 2). Monomeric BSA of 66 kDa as reported in the literature was expected in the control BSA sample. However, minor aggregation was observed in the unprocessed BSA (A1 and C2) as faint bands were observed in the high molecular weight region. Induced degradation and aggregation were shown as controls in the gel. Physical mixtures of BSA and HPβCD did not cause any noticeable protein instability. After SFD, no increase in protein degradation was noted than the unprocessed control, while aggregation was observed in all formulations. For the factorial design formulations, aggregation of BSA became more obvious, especially for ABC’ (B2), in which an intense band corresponding to high molecular weight was observed. The level of protein aggregation of the spray-freeze-dried formulations was further examined by SEC-HPLC (Figure 3). The general pattern of aggregation revealed by SEC was consistent with that of SDS-PAGE. All the spray-freeze-dried formulations displayed a higher level of aggregation than the unsprayed BSA. The result was also consistent with SDS-PAGE in that B2 displayed the highest level of aggregation. Interestingly, formulations that consisted of the lowest content of BSA (ABC, ABC’, AB’C and AB’C’) had the highest degree of aggregation, especially ABC’ and AB’C’, which were both prepared at a high atomization gas flow rate.

### 3.5. Factorial Design Analysis

**Figure 4:**
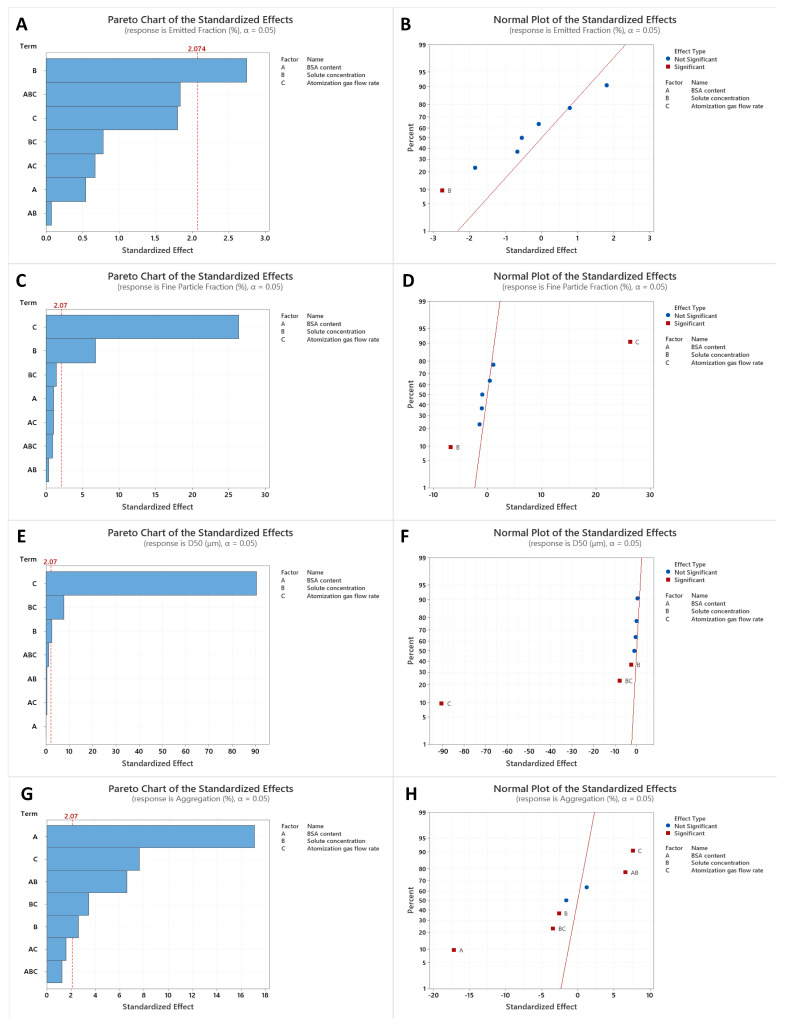
Factorial design analysis of the spray-freeze-dried powder formulations. Pareto charts (**A**,**C**,**E**,**G**) illustrate the importance of the independent variables and their interactions on EF (**A**), FPF (**C**), volumetric diameter (**E**), and protein aggregation (**G**). The factors that cross the vertical reference line indicate that the effects are statistically significant. Normal probability plots (**B**,**D**,**F**,**H**) illustrate the magnitude, direction, and importance of the independent variables and their interactions on EF (**B**), FPF (**D**), volumetric diameter (**F**), and protein aggregation (**H**). Effects that are further from 0 are more statistically significant. EF: emitted fraction; FPF: fine particle fraction.

The factorial analysis indicated that solute concentration has a significant effect on EF, with higher concentration causing lower EF (Figure 4A,B). Considering that all formulations had very high EFs of at least 90%, the effect of solute concentration was not crucial in this aspect, and all the spray-freeze-dried formulations prepared in this study exhibited excellent dispersibility. The FPF and volumetric diameter are important in determining the ability of the powder to deposit in the lower airways. The factorial analysis showed that both solute concentration and atomization gas flow rates are two important factors in influencing the site of lung deposition of these formulations (Figure 4C–F). High solute concentration had a negative impact as it resulted in a significantly lower FPF. This was accompanied by the barely significant positive effect on volumetric diameter. On the other hand, raising the atomization gas flow rate led to positive effects as doing so increased the FPF and decreased the volumetric diameter. The effect of atomization gas flow rate was consistent with the observations from the SEM images, which suggested that a high atomization gas flow rate led to the formation of smaller particles. When SFD was operated at a high flow rate of 670 L/h, the MMAD was below 1 µm, as demonstrated in ABC’ and A’BC’ formulations. When the aerodynamic diameter was too small (<1 µm), there is a possibility that the inhaled particles do not have sufficient time to settle before exhalation, thereby reducing delivery efficiency [21].

It is surprising to see that BSA content was identified as a significant factor in affecting protein aggregation after SFD in a reversed manner, i.e., the higher the BSA content, the lower the level of aggregation (Figure 4G,H). Since there were only two ingredients in the formulation, when the BSA concentration was increased, there should be less HPβCD available to protect the BSA. It appeared that those with a lower BSA content exhibited a higher level of aggregation. Apart from the BSA content, the atomization gas flow rate was also found to be a significant factor, with higher flow rates aggravating protein aggregation. It is anticipated that a high flow rate would result in more damage to the integrity of the protein due to high shear stress.

In view that a high atomization gas flow rate can generate particles that are in the appropriate size range (between 1 and 5 µm) for delivery into the deep lung [22], but would at the same time promote protein aggregation, the mid-level flow rate (i.e., 473 L/h) was identified as the optimal condition in this study. Since protein content was of great interest in understanding how the drug content may affect the formulation properties, extended formulations were, therefore, prepared based on the center point conditions (i.e., 5% solute concentration with an atomization gas flow rate of 473 L/h), while varying the BSA content from 0 to 100%.

### 3.6. Extended Formulations—The Effect of BSA Content

Interestingly, no significant difference was observed between the extended formulations of different concentrations of BSA in terms of morphology (Figure 1), particle size distribution and aerosol performance (Table 4). Indeed, all the extended formulations, which were prepared under the same conditions (solute concentration and atomization gas flow rate) as the center-point formulations, exhibited very similar properties to the three center point formulations (i.e., CP-1, CP-2, and CP-3). It appears that the physical and aerosol properties of spray-freeze-dried formulations were dominated by the operation parameters instead of the protein content. In terms of protein aggregation, all the extended formulations that contained HPβCD had a significantly lower level of aggregation compared with EXT-100, which did not consist of HPβCD, suggesting that the HPβCD did in fact, offer some level of protection to the BSA during SFD, albeit a rather mild effect. However, some degree of protein aggregation was still observed, with EXT-100 (C12) being the most obvious. In contrast to the factorial design formulations, as the amount of BSA increased (or HPβCD decreased), the level of protein aggregation appeared to increase as well, although differences between the formulations were unremarkable. Protein concentration-driven aggregation is foreseeable, given that the propensity for molecular interaction, which is a prerequisite for the formation of aggregates, is increased with a higher bulk protein concentration [23].

## 4. Discussion

A stable and effective delivery system is paramount to translate inhaled protein therapy into clinical use to treat respiratory diseases. SFD is a particle-engineering technique that deserves intensive investigation for the production of protein powders due to its relatively mild operating conditions. Solvent sublimation of atomized particles has often led to the formation of spherical, porous particles with low density, which promotes aerosolization [9]. Furthermore, recent developments in SFD technology have allowed production scale-up and continuous manufacture to become possible [5,24], making it a feasible method to manufacture protein therapeutics on an industrial scale. However, our understanding of SFD is still rather limited compared to other drying methods, such as SD and freeze-drying, partially due to its rather diverse approach in producing dry powder. Here, the relatively straightforward ‘spraying into vapor over a cryogenic liquid’ approach was used [10]. Three parameters, namely, protein content, solute concentration, and atomization gas flow rate, were investigated in this study to examine their effects on aerosol properties and protein stability, both of which are critical in determining the success of inhaled powder formulations of biotherapeutics.

According to the factorial design analysis, solute concentration and atomization gas flow rate were the two significant factors in affecting the aerosolization properties of the spray-freeze-dried powders, with the latter being the more dominant factor. These findings were consistent with previous studies where a high atomization gas flow rate reduced particle size [7,25], narrowing it to the range suitable for lung deposition. This was also reflected in the higher FPF values. Solute concentration also contributed to this effect, as the lower the solute concentration was, the more porous and thus less dense the particles became, improving airflow and facilitating powder aerosolization [26,27]. In contrast, solute concentration did not appear to have a major role, despite the statistical analysis, which indicated that it had a negative impact on powder dispersibility. Since all the spray-freeze-dried formulations exhibited superb EFs of over 90%, the effect was considered to be of minor relevance. The BSA concentration employed in the factorial design was rather low, with 10% *w*/*w* set as the high-level. A more substantial BSA content is necessary to gain a better understanding of how it may affect powder properties.

To investigate the effects of BSA content across a wider range, extended formulations were designed and prepared. The center point parameters were chosen instead of the low solute concentration and high atomization gas flow rate, which showed the highest FPF according to the factorial analysis. This was because the spray-freeze-dried powders obtained under these conditions (ABC’ and A’BC’) had MMAD of less than 1 µm, which could lead to a smaller proportion of powders being deposited properly in the lung [22]. More importantly, the high atomization gas flow rate was a significant factor contributing to protein aggregation. Moreover, if the solute concentration is too low, the particles may be too fragile and create debris during powder dispersion, which could be seen under an SEM. On the other hand, the center point formulations still displayed a good FPF of about 60% with MMAD of around 1.9 µm, underlining the desirability of the parameters for further investigation.

Interestingly, all the extended formulations exhibited very similar aerosol performance in terms of EF, FPF, and MMAD, despite stretching the BSA content from 0 to 100%. Their morphology under SEM was almost undifferentiated. A trend was observed in that increasing BSA content led to a decrease in FPF, although there was no significant difference among these samples (*p* > 0.05). Either HPβCD and BSA shared certain similar characteristics so that their relative contents in the formulations did not influence powder dispersion and aerosolization properties, or SFD is indeed such a robust drying method that the aerosol properties are largely determined by the operating conditions rather than the formulation. This prompts the need to incorporate different excipients in future work to corroborate these claims.

It is known that protein therapeutics are susceptible to physical degradation, especially when they are in a liquid state. By formulating protein into a solid form, part of the problem has already been circumvented. Yet, during SFD, protein molecules are inevitably exposed to shear (during atomization), interfacial (air bubble entrapment during atomization shear), dehydration, and thermal stresses, which may result in irreversible degradation, denaturation, aggregation and fragmentation [12,28,29]. Protein aggregation is the most notorious type of protein instability, and it can provoke immunogenicity and exacerbate the loss of efficacy [22,30]. The observation that increasing BSA content reduced the degree of aggregation, notably between 2% and 10% BSA, could be explained by the volume exclusion effect of macromolecular crowding. This hypothesis proposes that an increased amount of macromolecule solutes suppresses unfolding and reduces overall protein mobility during spray-freeze-drying. Such a macromolecular crowding effect is thermodynamically stabilizing [31,32]. Another rationale to explain the observation is finite interfacial adsorption, where protein aggregation manifests at the interface between air and water during spraying or between ice and water during freezing [33]. When the interface is saturated with protein molecules, any increase in the concentration of the protein in bulk will reduce the relative proportion of aggregated proteins [34]. The exact mechanism of such a phenomenon in our formulations could be explored.

To minimize protein-protein interactions and thus aggregation, stabilizers are needed in the formulation. They act through water replacement and vitrification mechanisms of stabilization [11]. Here, HPβCD could prevent the fragmentation of BSA, as observed in the extended formulations. However, it could not completely protect the protein from aggregation, indicating that the formulations need to be further improved, possibly by including other stabilizers and/or optimizing the SFD parameters [35]. Taking into account the possibility of consequential shear and interfacial stresses associated with SFD that sugars might not be adequately effective against, surfactants would be a valid class of excipients that should be considered for inclusion in future formulations [12], particularly when a high atomization gas flow rate is desired. The surfactant-like behavior of HPβCD [18] could plausibly be augmented by the addition of a surfactant at low concentrations, such as polysorbate 80 [36], which is already approved by the US FDA for the inhalation route. Nevertheless, this study has demonstrated that HPβCD is a viable excipient in the preparation of spray-freeze-dried protein formulations with good aerosol properties, notwithstanding the limited stabilizing effect.

## 5. Conclusions

The evaluation of the feasibility of dried powders containing biologics intended for inhalation therapy encompasses the assessment of the aerosol performance and protein stability. In this study, through factorial design analysis, high atomization gas flow rate was identified as a significant operating condition that enhanced aerosolization properties of the spray-freeze-dried powders but also promoted protein aggregation. All the powder formulations displayed superb dispersibility, and the BSA was protected against fragmentation. Extended formulations based on the center point conditions did not highlight any influence of BSA content on aerosolization properties or protein aggregation. However, some appreciable increase in aggregation still occurred despite the presence of HPβCD, which suggests that the formulation needs to be further optimized.

## Figures and Tables

**Table 1:** The 2^3^ full factorial experimental design for spray-freeze-dried powder formulations. The three levels of each factor were designated as −1 (low level), 0 (middle level), and +1 (high level).

Factor	Level		
	−1	0	+1
A—BSA content (% *w*/*w*)	2	6	10
B—Solute concentration (% *w*/*v*)	2.5	5	7.5
C—Atomization gas flow rate (L/h)	301	473	670

**Table 2:** Formulations of the spray-freeze-dried powders. CP: center point; EXT: extended formulation; an apostrophe denotes high-level factor.

Sample	A—BSA Content (% *w*/*w*)	B—Solute Concentration (% *w*/*v*)	C—Atomization Gas Flow Rate (L/h)
2^3^ full factorial design formulations			
ABC	2	2.5	301
ABC’	2	2.5	670
AB’C	2	7.5	301
AB’C’	2	7.5	670
A’BC	10	2.5	301
A’BC’	10	2.5	670
A’B’C	10	7.5	301
A’B’C’	10	7.5	670
CP-1	6	5	473
CP-2	6	5	473
CP-3	6	5	473
Extended formulations			
EXT-0	0	5	473
EXT-25	25	5	473
EXT-50	50	5	473
EXT-75	75	5	473
EXT-100	100	5	473

**Table 3:** Production yield and composition of the spray-freeze-dried powders.

Sample	Production Yield (%)	BSA Content (%)	HPβCD Content (%)
ABC	84.4	N.A.	102.7 ± 5.3
ABC’	77.3	N.A.	96.9 ± 2.0
AB’C	75.3	N.A.	100.0 ± 1.3
AB’C’	72.8	N.A.	95.7 ± 4.4
A’BC	78.5	N.A.	88.6 ± 4.5
A’BC’	72.6	N.A.	86.4 ± 5.2
A’B’C	76.0	N.A.	93.4 ± 1.8
A’B’C’	71.5	N.A.	91.6 ± 3.6
CP-1	71.0	N.A.	97.4 ± 2.3
CP-2	71.8	N.A.	93.5 ± 7.4
CP-3	73.0	N.A.	96.3 ± 5.7
EXT-0	91.1	0	94.8 ± 2.4
EXT-25	93.8	27.4 ± 0.3	71.3 ± 0.2
EXT-50	97.4	52.8 ± 0.2	46.8 ± 2.0
EXT-75	97.6	76.0 ± 0.7	23.0 ± 0.1
EXT-100	88.6	96.0 ± 0.5	0

N.A.: not applicable. The content of BSA in the formulation was too low (<10%). Hence the content of HPβCD was quantified instead.

**Table 4:** Particle size distribution and aerosol performance of spray-freeze-dried powder. The volumetric diameter of the particles was measured by laser diffractometry. D_10_, D_50_, and D_90_ represent the equivalent spherical volume diameters at 10%, 50%, and 90% cumulative volumes, respectively. Aerosol performance was evaluated using a next-generation impactor (NGI). The emitted fraction (EF) refers to the fraction of powder that exited the inhaler; the fine particle fraction (FPF) is defined as the percentage of particles with aerodynamic diameter below 5 µm. Mass median aerodynamic diameter (MMAD) was calculated based on the NGI data. The data are presented as mean ± standard deviation.

Formulation	Volumetric Diameter				EF (%)	FPF (%)	MMAD (µm)
	D_10_ (µm)	D_50_ (µm)	D_90_ (µm)	Span			
ABC	11.2 ± 0.2	30.2 ± 0.9	58.6 ± 1.6	1.6 ± 0.0	96.0 ± 0.78	23.0 ± 2.6	9.0 ± 1.8
ABC’	4.1 ± 0.0	9.3 ± 0.2	20.2 ± 1.3	1.7 ± 0.1	96.0 ± 1.2	78.4 ± 1.1	1.0 ± 0.2
AB’C	6.7 ± 0.3	31.4 ± 1.1	60.0 ± 2.1	1.70 ± 0.0	93.4 ± 1.1	9.8 ± 2.5	13.6 ± 1.9
AB’C’	2.8 ± 0.1	7.1 ± 0.2	17.4 ± 0.7	2.1 ± 0.0	95.9 ± 1.4	63.1 ± 3.6	1.9 ± 0.3
A’BC	10.8 ± 0.2	29.9 ± 1.0	57.9 ± 2.0	1.6 ± 0.0	95.2 ± 1.1	24.6 ± 4.7	8.2 ± 4.2
A’BC’	4.3 ± 0.1	9.8 ± 0.1	21.3 ± 0.4	1.8 ± 0.0	96.3 ± 0.2	79.4 ± 1.0	1.0 ± 0.1
A’B’C	6.5 ± 0.2	31.4 ± 1.1	59.1 ± 1.4	1.7 ± 0.0	94.3 ± 2.5	16.7 ± 4.1	6.8 ± 1.2
A’B’C’	2.8 ± 0.1	6.8 ± 0.1	15.9 ± 0.9	1.9 ± 0.1	94.4 ± 1.5	62.1 ± 3.6	1.9 ± 0.3
CP-1	4.3 ± 0.1	10.8 ± 0.3	24.7 ± 1.0	1.9 ± 0.0	96.5 ± 0.5	60.4 ± 6.4	2.1 ± 0.5
CP-2	4.3 ± 0.1	10.8 ± 0.2	24.8 ± 0.3	1.9 ± 0.0	95.4 ± 0.9	59.8 ± 8.1	2.0 ± 0.8
CP-3	4.3 ± 0.0	10.6 ± 0.1	24.4 ± 0.3	1.9 ± 0.0	95.7 ± 1.1	61.1 ± 8.7	1.5 ± 0.2
EXT-0	3.9 ± 0.1	9.9 ± 0.3	24.4 ± 0.9	2.1 ± 0.0	98.5 ± 0.6	60.5 ± 2.7	1.8 ± 0.2
EXT-25	4.0 ± 0.0	10.4 ± 0.2	25.5 ± 0.5	2.1 ± 0.0	100 *	65.5 ± 1.7	1.4 ± 0.0
EXT-50	4.1 ± 0.0	11.1 ± 0.3	27.2 ± 1.2	2.1 ± 0.1	100 *	63.6 ± 1.8	1.4 ± 0.1
EXT-75	3.9 ± 0.1	10.8 ± 0.7	27.2 ± 1.9	2.2 ± 0.0	98.3 ± 0.7	54.7 ± 3.3	2.1 ± 0.3
EXT-100	3.8 ± 0.0	11.7 ± 0.5	31.1 ± 2.0	2.3 ± 0.1	98.4 ± 0.4	52.3 ± 4.7	2.5 ± 0.6

* The amount of protein in the capsule and inhaler was below the lower limit of the standard curve (i.e., unrecoverable from the capsule and inhaler).

## Data Availability

Not applicable.
